# Climate impacts on long-term silage maize yield in Germany

**DOI:** 10.1038/s41598-019-44126-1

**Published:** 2019-05-21

**Authors:** Michael Peichl, Stephan Thober, Luis Samaniego, Bernd Hansjürgens, Andreas Marx

**Affiliations:** 1 UFZ-Helmholtz Centre for Environmental Research, Department Computational Hydrosystems, Permoserstrasse 15, D-04318 Leipzig, Germany; 2 UFZ-Helmholtz Centre for Environmental Research, Department Economics, Permoserstrasse 15, D-04318 Leipzig, Germany

**Keywords:** Climate-change impacts, Climate-change impacts, Hydrology, Natural hazards

## Abstract

In this study, we examine the impacts of climate change on variations in the long-term mean silage maize yield using a statistical crop model at the county level in Germany. The explanatory variables, which consider sub-seasonal effects, are soil moisture anomalies for June and August and precipitation and temperature for July. Climate projections from five regional climate models (RCMs) are used to simulate soil moisture with the mesoscale Hydrologic Model and force the statistical crop model. The results indicate an average yield reduction of −120 to −1050 (kilogram/hectare)/annum (kg ha^−1^ a^−1^) for the period 2021–2050 compared to the baseline period 1971–2000. The multi-model yield decreases between −370 and −3910 kg ha^−1^ a^−1^ until the end of the century (2070–2099). The maximum projected mean loss is less than 10% in magnitude of average yields in Germany in 1999–2015. The crop model shows a strong ability to project long-term mean yield changes but is not designed to capture inter-annual variations. Based on the RCM outcomes, July temperature and August soil moisture anomalies are the main factors for the projected yield anomalies. Furthermore, effects such as adaptation and CO_2_ fertilization are not included in our model. Accounting for these might lead to a slight overall increase in the future silage maize yield of Germany.

## Introduction

There is growing evidence that all areas of daily life will be affected by climate change and that, in addition to existing initiatives for climate change prevention, adaptation measures are becoming increasingly necessary. One of the sectors exposed to the greatest risk of climate change is agriculture, as changes in meteorology and trace gas concentrations have direct impacts on crop yields and agricultural ecosystems^1^. While higher CO_2_ concentrations, higher average temperatures and longer growing seasons can have positive effects on crop yields, drought, heat stress, heavy rainfall and high ozone concentrations can reduce these yields^1^. A higher variability of individual weather events is expected^1^ because climate change not only increases temperature but also changes in precipitation patterns in space and time^2^. Data from a recent study show that the time under drought conditions in Germany will increase by approximately 50% with a global warming of 3 °C^3^. This variability is particularly relevant for agricultural production, as the sensitivity of plant growth to meteorological variations is time-dependent^4^. In this study, the impact of climate change on rainfed silage maize in Germany is examined, which is becoming increasingly important in the wake of the German *Energiewende* (energy transition) due to the increased demand for biomass.

It is necessary to know the impacts of climate change and what is causing these to provide sound recommendations for action. Within this context, there are two research communities that employ different tools to estimate crop yield, namely, process-based and statistical models. An explanation for the occurring differences in the results of the approaches are, among others, the factors used in the individual modelling approaches^5^. In this context, a particular problem with statistical models is proneness to collinearity. Apparently causal associations of weather determinants with yield variations can obscure underlying physiological mechanisms^6^. For example, the influence of heat on crop yields has not been fully clarified. This topic is important because the measure of extreme temperature over the entire growing season is often used as the main determinant of yield variation in statistical approaches while neglecting proper control for water supply. Accounting for plant water availability in a statistical approach leads to a reduced temperature sensitivity for silage maize yields in Germany^4^. A recent study^5^ concluded that harnessing the best features of both approaches can improve predictive power. Sub-seasonal patterns of precipitation, vapor pressure deficit, and solar radiation are implemented in process-based models but are often simplified or neglected in statistical approaches^6^. It is likely that aggregated measures of water supply commonly used in statistical models, such as precipitation averaged over the entire growing season, have lower sensitivities than those found in process-based models because seasonal effects and extremes can be averaged out^7^.

Recently, a meta-analysis on climate impacts for central Europe projected a change in average maize yield of −9% for the 2020 s and −15% for the 2080 s^8^. Literature on impact assessments for parts of Germany based on aggregated time series models with estimates at the district level under the A1B scenario show moderately negative effects on maize for East Germany by the middle of the 21^*st*^ century, moderately negative to positive effects on maize for Saxony-Anhalt, and positive effects on maize for North Rhine-Westphalia^9–11^. Negative impacts on silage maize are mainly found with a global increase in temperature of 3 °C for the East German plains^12^. No consistent assessment for entire Germany is currently available.

In this study, we examine the impacts of climate change on variations in the long-term mean of silage maize yield for all counties in Germany. A reduced-form model is developed and fitted for the period 1999–2015 for which yield records on county level are available. We explicitly use the most relevant factors of a statistical model, which considers sub-seasonal variations of meteorological variables and soil moisture anomalies to predict silage maize yields (hereafter PTMS)^4^. Those are dry and wet soil moisture anomalies for June and August and temperature and precipitation for July. The soil moisture anomalies are calculated as an index^13^ and are based on the output of the mesoscale Hydrologic Model (mHM)^14^. Climate simulations only show robust trends with rough temporal resolutions. Therefore, we argue that the persistence of soil moisture and the resulting smoother distribution compared to the meteorological variables can provide a more reliable climate assessment compared to those based only on meteorological variables (see Supplementary Fig. S1 for more information)^4^. Extreme annual yield variations, e.g., due to drought, are not explicitly considered in this study. Five hydro-meteorological simulations are used to force the statistical crop model. Changes in the long-term average crop yield are evaluated for two climate periods (2021–2050 and 2070–2099) compared to the reference period 1971–2000.

## Results and Discussion

### Estimated coefficient of the regression model

The coefficients estimated by the reduced-form model combining the major hydro-meteorological predictors closely match those found in PTMS^4^. The largest effects estimated for soil moisture are −52 decitonnes/hectare (dt ha^−1^ = 100 kg ha^−1^), which is about −11.6% for severely wet soil moisture conditions in June and 47 dt ha^−1^ (−10.5%) for severe drought conditions in August, all other determinants being equal (Table 1). We would like to stress that the SMI is monthly percentile-based index. The SMI in June and August corresponds to different soil water saturation fractions (for various locations in Germany, the annual development of soil moisture fractions are shown in Fig. 4 in Samaniego *et al*.^13^). In June, wet anomalies represent potentially harmful soil moisture above optimal conditions. The soil has been replenished in the past seasons, and a high level of moisture saturation in the soil can, for example, lead to water logging or luxury consumption and thus to lower root depth. From July, the soil water content decreases below the optimal conditions (60–80% of the available field capacity)^15^. As a result, dry anomalies represent harmful conditions because the available soil water is too low to provide enough water in the most drought-susceptible phases of flowering, pollination and grain filling^1^. These results highlight that the availability of water is key for the successful cultivation of arable crops in Germany. Soil moisture is considered a major limiting factor to simulated crop yields, in particular during sensitive phenological stages^16^.

**Table 1:** Table of Regression.

Variable	Month	Specification	Silage Maize Anomaly SE: (Standard)/(Driscoll-Kraay)/(Bootstrap)
Precipitation	July	Polynom (degree 1)	0.264*** (0.028)/(0.033)/(0.110)
	July	Polynom (degree 2)	0.001 (0.0003)/(0.001)/(0.001)
	July	Polynom (degree 3)	−0.00001** (0.00000)/(0.00000)/(0.000)
Temperature	July	Polynom (degree 1)	−6.443*** (0.634)/(1.001)/(5.840)
	July	Polynom (degree 2)	−4.050*** (0.305)/(0.291)/(3.664)
	July	Polynom (degree 3)	0.703*** (0.078)/(0.104)/(0.976)
Soil Moisture Index	June	severe drought	10.622*** (2.196)/(2.880)/(7.150)
	June	moderate drought	8.723*** (1.988)/(2.303)/(3.960)
	June	dry	3.198* (1.722)/(1.763)/(2.561)
	June	wet	−6.155** (2.203)/(2.462)/(4.311)
	June	abundantly wet	−12.173*** (2.660)/(3.813)/(5.767)
	June	severely wet	−52.091*** (3.618)/(5.850)/(21.034)
Soil Moisture Index	August	severe drought	−47.447*** (2.609)/(3.820)/(12.549)
	August	moderate drought	−21.952*** (2.066)/(2.837)/(5.985)
	August	dry	−8.200*** (1.771)/(2.495)/(2.716)
	August	wet	0.656 (2.084)/(1.800)/(4.000)
	August	abundantly wet	−3.447 (2.428)/(2.431)/(5.881)
	August	severely wet	−10.703*** (3.548)/(3.755)/(10.706)
Constant			18.905*** (1.155)/(1.527)/(4.710)
In-sample:	R^2^: 0.389	Adj. R^2^: 0.387	Adj. R^2^ - full variation: 0.705
LOCV (10-folds, 20 repeats):	R^2^: 0.385	RMSE: 37.014	MAE: 28.288
LOCV (annual blocks):	R^2^: 0.083	RMSE: 39.145	MAE: 30.589
LOCV (state blocks):	R^2^: 0.378	RMSE: 37.637	MAE: 29.169
Observations	4,625		

Standard errors are derived from three configurations. The first is the standard parametric configuration, and the second is the Driscoll-Kraay standard error, which parametrically accounts for serial and cross-sectoral autocorrelation and heteroscedasticity. The third configuration is based on a bootstrap approach resampling the years in the sample. The smallest standard errors are reported by the standard configuration, and the largest standard errors are reported by the bootstrap configuration.

Note: *p < 0.1; **p < 0.05; ***p < 0.01; based on Driscoll-Kraay Standard Errors.

The in-sample adjusted coefficient of determination is 0.38 (Table 1). However, when comparing this estimate with the results of other studies, it should be noted that the model used here only accounts for inter-annual variation. A model that uses the full crop yield variation and fixed effects has an adjusted R^2^ of 0.71 (Table 1). The out-of-sample fit measures, which were derived from leave-out cross validation, are comparable to the in-sample measure, except for when annual blocks were omitted. For the latter resampling approach, the coefficient of determination decreases, while other out-of-sample measures such as root mean squared error (RMSE) and mean absolute error (MAE) only slightly increase. The reason for this result is assumed to be the higher sensitivity to outliers of the coefficient of determination than of the RMSE and MAE, which may be due to the relatively short silage maize yield record of 17 years.

### Model evaluation against historical observations

**Figure 1:**
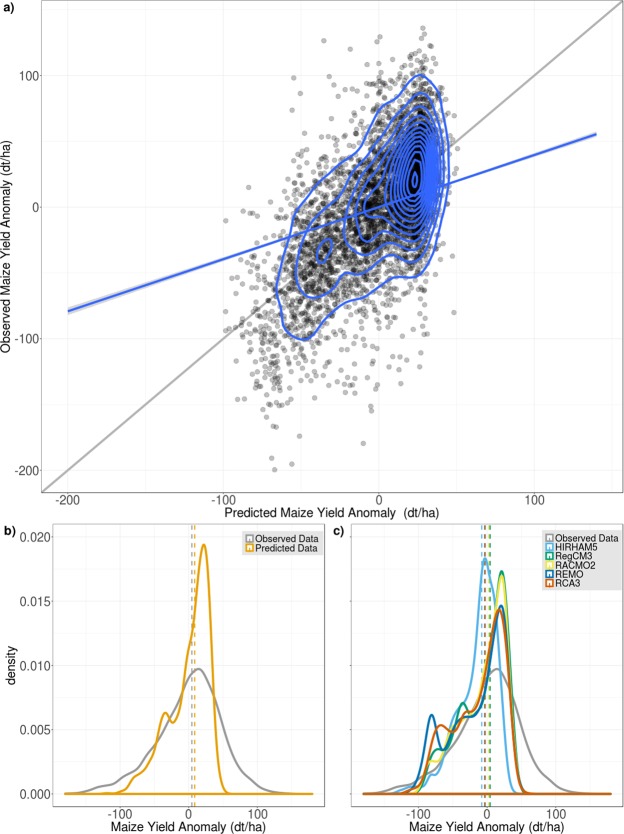
Scatterplot and density plots of the observed maize yield anomaly data against the simulated data. In panel (a) the observed data (Y-axes) are plotted against the predicted yield anomaly data (X-axes) for the period 1999–2015. The blue contour lines show the density of the point cloud, and the blue line shows the linear fit. Panel (b) shows the marginal density of the observed and the predicted data (derived from observed meteorological forcings) for the period 1999–2015. In panel (c), the observed data are compared against the projected data with input data derived from the 5 different regional climate models for the period 1999–2015. The dashed lines in the density plots represent the median of each distribution.

**Figure 2:**
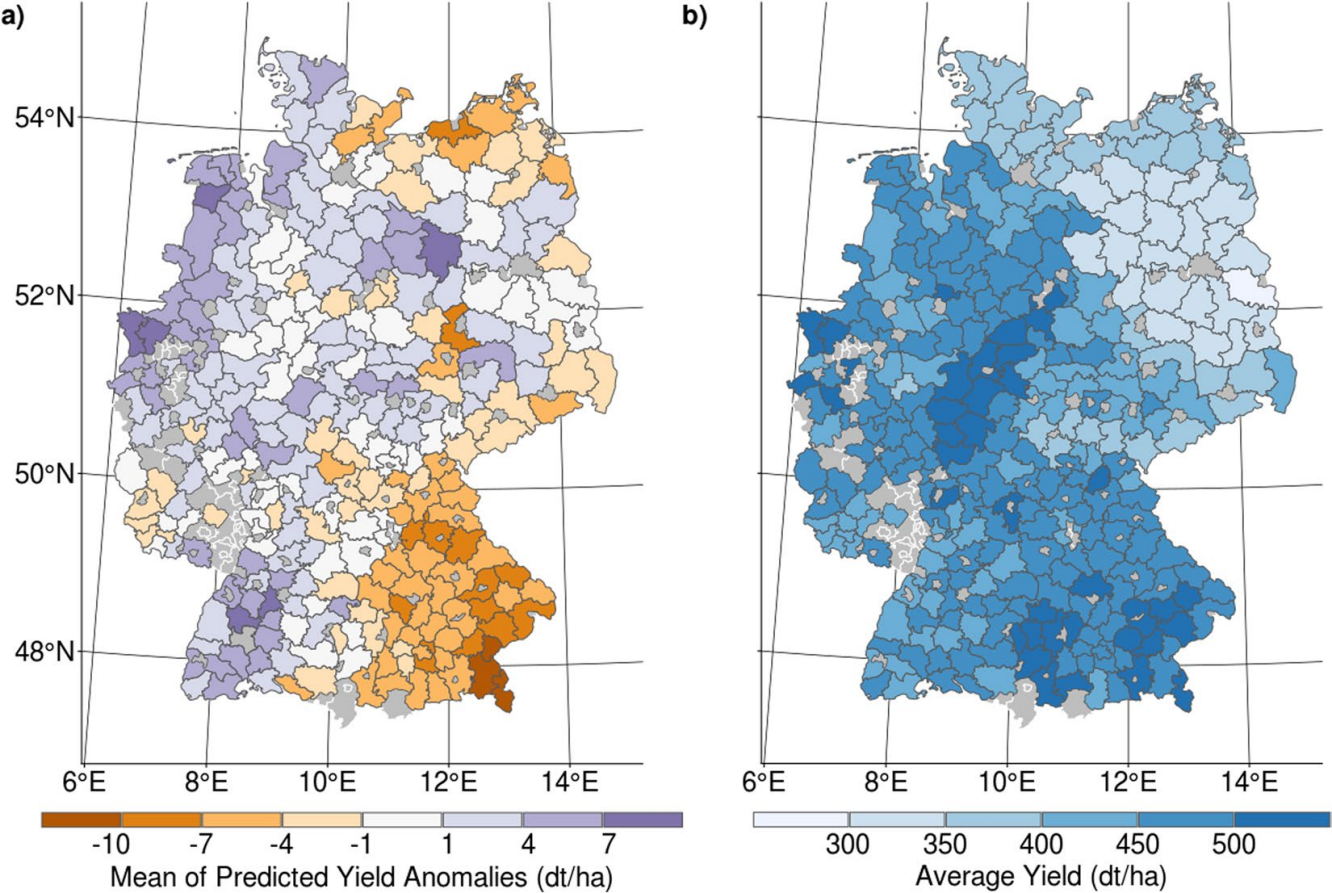
The map in panel (a) shows the difference between the average predicted and actual yield anomalies at the county level for the period 1999–2015. Panel (b) shows the average yield of each county for this period. Grey areas indicate the counties neglected in the model due to insufficient sample sizes. See Supplementary Figs S2 and S3 for further information.

There is a large difference between the observed and predicted yield anomaly data (Fig. 1a). The range of the observed anomalies is between −200 and 144 dt ha^−1^, and the range of the predicted anomalies is between −120 and 55 dt ha^−1^. As the density contour lines show, the data around the mode are better predicted than the extreme values (Fig. 1b). In general, the variability of the data is underestimated by the model, mostly because positive yield deviations are not captured by the model. However, the model is able to predict the observed values over the entire period. The long-term difference between the predicted and actual yield anomalies for the period 1999–2015 is between −13 and 10 dt ha^−1^ (Fig. 2). The relative deviation is at most 2.36% for each county (Fig. 2b).

We also evaluated the model using the hydro-meteorological data derived from the regional climate models (RCMs) for the period 1999–2015. The model overall underestimates the observed values over Germany for the individual RCMs in similar ways as this is the case with historical data as input (Fig. 1c). The median values of the simulations using input data derived from the RCMs (dashed lines) are slightly below the median of the observations. The shape of the distributions of the simulations differ from the distribution of the observations mainly in the negative range. However, the negative estimates reflect the bandwidth of the observed data better than that of the positive range. This result indicates that the approach is not able to capture positive extremes and overestimates negative climate impacts. The long-term district averages for near- and far-future periods in comparison to averages of the reference period 1971–2000 are compared in the following subsection.

### Climate projections

**Figure 3:**
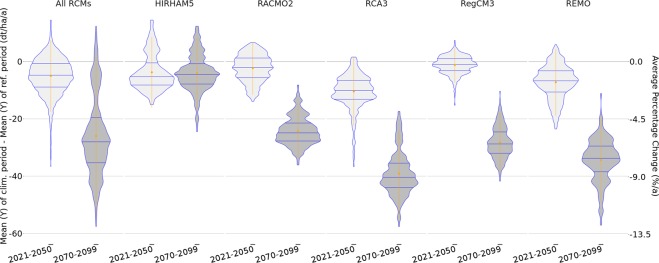
Violin plot of the projected average yield anomalies at the county level for the periods 2021–2050 and 2070–2099 compared to the reference period 1971–2000. The first panel shows the cumulated results for all RCMs, and the other five panels show the results for each RCM separately. The blue lines represent the quantiles 0.25, 0.5, and 0.75. The orange dots show the mean values, and the vertical lines emanating from each dot represent the standard error times 2.

The variation in the average yield anomalies of silage maize was estimated for the reference period (1971–2000) and two climate periods (near future: 2021–2050 and far future: 2070–2099). Five RCMs (HIRHAM5, RegCM3, RACMO2, REMO, RCA3) were used to drive the mHM and the statistical crop model. All RCMs project decreases in silage maize yield. The average projections for all five multi-model simulations are −5 dt ha^−1^a^−1^ (≈−1.1% a^−1^) for the near future period and −25 dt ha^−1^a^−1^ (≈−5.6% a^−1^) for the far-future climate period (Fig. 3). There is a consensus that decreases in yield will be larger in the second half of the 21^st^ century than in the near future, with less severe damages in regions with temperate climates^17,18^. These results are confirmed by the changes in average yield presented in Fig. 3 because all RCMs exhibit a lower magnitude of change in the near future period than in the far-future period.

In this study, the biophysical processes in the statistical models are approximated by incorporating measures of sub-seasonal soil moisture anomalies, which are assumed to support the convergence in the outcomes between the statistical models and process-based models^4,7,19^. Other factors reflected in process-based models that are usually neglected in statistical models are the effects of adaptation, CO_2_ fertilization, and ozone^7^. The impact of the first two factors will be discussed here, while the last factor will not be considered because there is a lack of scientific understanding of the effects of ozone. First, adaptation in process-based models is sometimes referred to as ’adaptation illusion’^20^ because it usually only represents on-farm or within-crop adaptation that provides benefits unconditional on climate development^21^. For instance, global computable general equilibrium models specifically designed for the agricultural sector could contribute to truly account for economic adaptation^21^. Second, CO_2_ fertilisation can explain more variability in the agricultural sector as for instance adaptation^5,21^. For this reason, it should be taken into account when the impact assessment using statistical approaches is evaluated^1,7,21^. The CO_2_ fertilization effect can, among other ways, be considered using a yield correction model^9^. Since Maize is a C4 plant it mainly benefits from the increase in CO_2_ under drought conditions through reduced transpiration as long as nitrogen supply is not limited^22,23^. The correction factors therefore consider both the rather negligible direct yield effect through stimulated photosynthesis and the more important compensation of yield losses from drought stress through increased water use efficiency by reducing the stomatal conductance^24,25^. Both are a function of CO_2_ change and translate yield projections without CO_2_ fertilization into estimates with CO_2_ fertilization. Accordingly, an estimated yield change without CO_2_ fertilization of −10% can be transformed to an estimated yield change of +5% by 2056 and +11% by 2086 for the CO_2_ levels in the A1B scenario^10^. In the study presented here, the highest projected average yield loss (RCA3 in the second climate period) is less than −10% in magnitude. As explained later for the five regional climate models considered here, factors related to dry conditions such as temperature in July and soil moisture deficit in August usually correlate with yield variability. Thus, when assuming that rising CO_2_ will benefit maize growth under drought conditions^22,23^, slightly positive yield changes may be expected on average even without taking into account potential adaptation. The approach in this study has several limitations. It is assumed that the currently known connections will continue in the future because the impact model is trained with historical data. Thus, the approach is not able to take into account future developments not reflected in the past^12^. Extreme climate anomalies are scientifically accepted to be a consequence of climate change and are known to have significant impacts that pose elementary adaptation and economic challenges to farmers^17,26,27^. These effects are, for instance, linked to the duration, area and frequency of droughts^3^. Simultaneous production shocks related to silage maize caused worldwide by climate change are also not taken into account^28^. The analysis in this study is focused on mean yield changes and does not assess the climate-induced year-to-year variability of crop yields, e.g., large losses caused by droughts from which farmers are not able to recover. This increases the uncertainty in our results, especially for the second half of the century^1^. Here, only the variance in the long-term means of climate periods is assessed. The projected variance of the mean yield losses is between −36.7 dt ha^−1^ a^−1^ and 14.5 dt ha^−1^ a^−1^ for the first period and between −57.6 dt ha^−1^ a^−1^ and 12.4 dt ha^−1^ a^−1^ for the second period. The upper boundaries of the variations are marked in both climate periods by HIRHAM5, and the lower boundaries are marked by RCA3. There are high inter-model variabilities in the projected averages of the mean yield losses. The smallest values in the mean yield losses are generally projected by RegCM3 (−1.2 dt ha^−1^ a^−1^) in the first period and by HIRHAM5 (−3.7 dt ha^−1^ a^−1^) in the second period. In both climate periods, RCA3 generally projects the highest mean yield losses (−10.5 and −39.1 dt ha^−1^a^−1^). This variability, however, mainly reflects the spatial heterogeneity of the projected mean yield losses.

### Influence/spatial analysis of individual regional climate models

**Figure 4:**
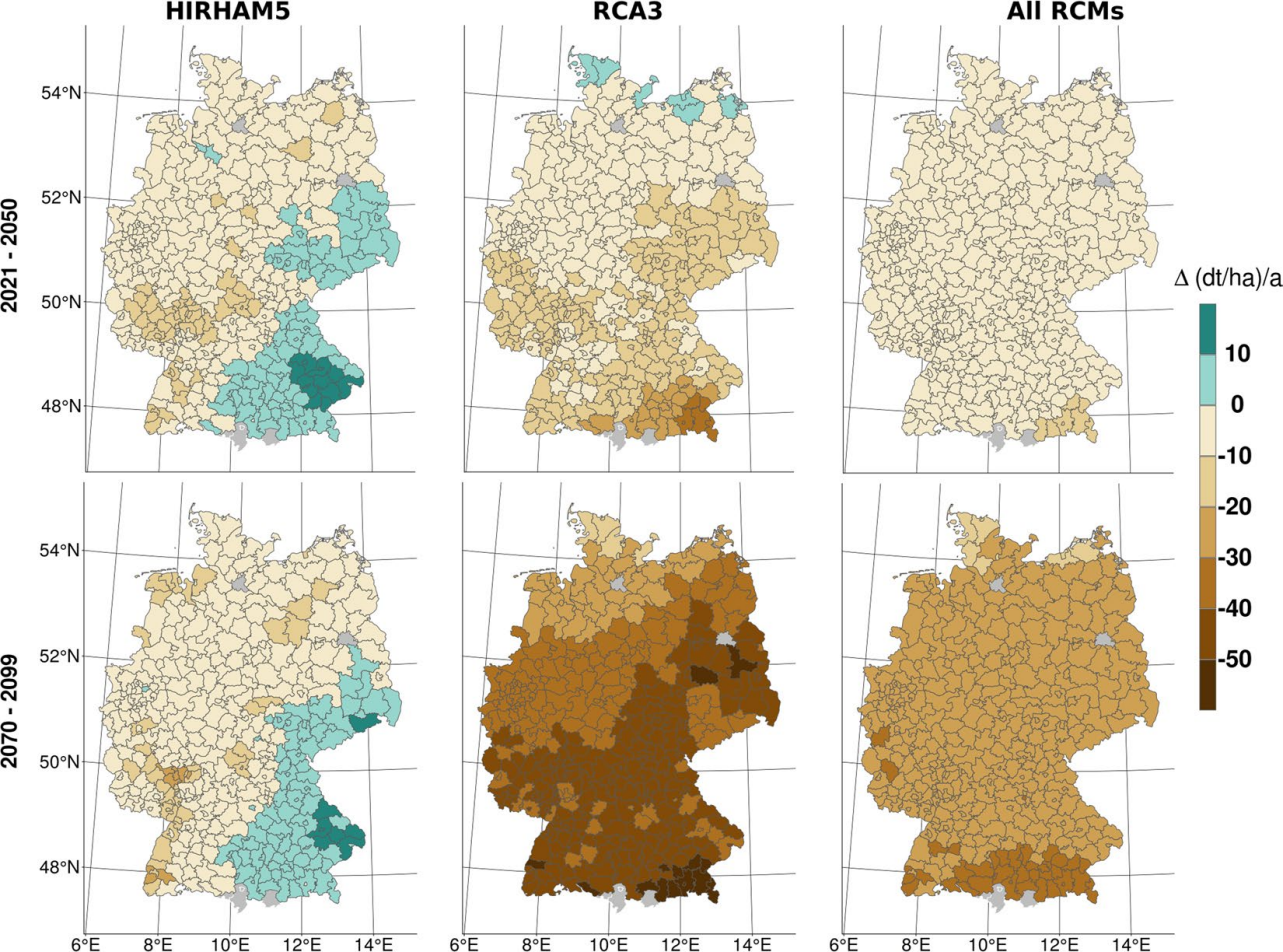
Selected maps of county-specific yield anomaly deviations (climate period-reference period) for both climate periods. The first column represents the lowest average yield anomaly deviations (derived by HIRHAM5), the second column the highest average yield deviations (RCA3), and the third column shows the county-specific mean of all yield anomaly deviations projected by the five RCMs for each county. The first row represents the climate period 2021–2050, and the second row represents the climate period 2070–2099.

The spatial patterns in the mean yield anomaly differ among the RCMs (Fig. 4). There are also differences in the mean yield anomaly spatial patterns between the climate periods. Projected yields based on the HIRHAM5 model (column 1 of Fig. 4) increase in south-east Germany, while small decreases are projected by the other RCMs in this region. This model predicts the lowest mean losses overall. Decreasing yields are projected by the RCA3 model during both future periods along a gradient from north-west to south-east Germany. These decrements are larger for the second climate period than the first climate period. This trend also applies to estimates derived from all other models except for HIRHAM5 (Fig. 3). As shown previously, other projections for the east of Germany show a negative future yield development, while a positive future yield development is predicted for the west of Germany^9–12^. These studies use time series approaches for each district, allowing more flexible yield sensitivities to external meteorological and soil variations. However, there are several reasons in support of a panel approach. First, this approach is less susceptible than other approaches to coefficient bias caused by omission of time-invariant factors. Second, we can only evaluate the reported yield data for each district for a 17-year time period. A panel approach increases the data set by considering the time series and spatial information from counties.

The multi-model ensemble mean exhibits very little spatial heterogeneity, with slightly higher losses in the south of Germany than in other areas (Fig. 4, column 3). Since the impact model takes into account different sensitivities to different factors over the season, it responds to certain patterns reproduced by the RCMs. Thus, the projected yield estimates cancel each other out when averaged in a multi-model ensemble.

**Figure 5:**
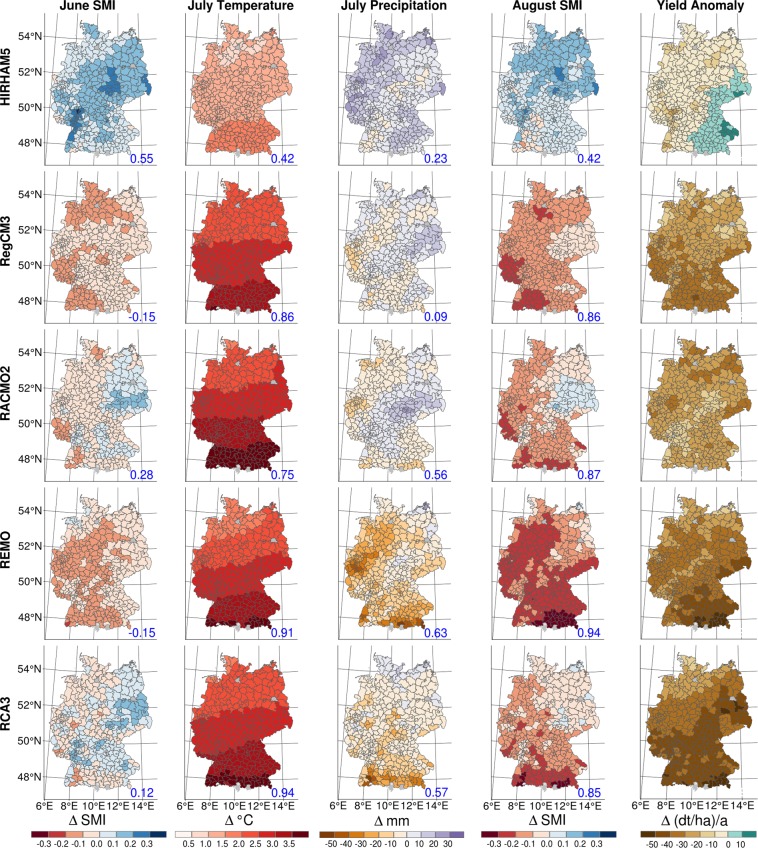
All panels show maps with the mean value changes within individual counties, with either explanatory variables or yield anomalies derived from the various RCMs for the second climate period (2070–2099). The columns represent the different variables, and the rows represent the RCMs (HIRHAM5, RegCM3, RACMO2, REMO, RCA3). The explanatory variables are normalized by the procedure used for yield anomalies. The blue numbers indicate the Spearman correlation coefficients of the mean data. A more detailed description can be found in the Supplementary Information.

Figure 5 shows maps of the mean changes for the second climate period (2070–2099) within each county, for both the predictors and the yield anomalies (descriptive statistics can be found in the Supplementary Table S1). Different patterns in SMI and meteorological changes can be observed among the individual RCMs, with HIRHAM5 exhibiting the most distinct patterns. For example, in June, the SMI shows a broad range of changes in all five RCMs (first column). HIRHAM-driven simulations show that the soil moisture index increases comparatively over time, while RegCM3 and REMO show a decrease in future soil moisture represented by the index. For the other RCMs, a mixed development is shown. Overall, the long-term mean changes in SMI are between −0.19 (RCA3 and RegCM3) and 0.31 (HIRHAM5) in June.

As expected, the maps in the second column of Fig. 5 show an increase in temperature in July for all RCMs. In addition, the spatial temperature trends show greater increases in the south than in the north. For HIRHAM5, the model with the lowest temperature increase, the maximum increase is 2 °C. For REMO, the model with the second lowest temperature rise, the maximum increases are between 1.7 °C and 3.7 °C; for the other RCMs, the maximum increases range between 2 °C and 4 °C.

Notably, annual temperature fluctuations are not sufficient to explain the development of crops. In fact, the temperature changes in the periods in which plant development is particularly susceptible to heat, such as the reproductive, flowering, and grain-filling stages, are most important^25,29–31^. Heat can, for example, shorten the grain-filling phase and thus lead to a reduction in yield and quality. However, the susceptibility of plants to heat, especially silage maize, is reduced by an adequate water supply^32^. For maize, only temperatures above 35 °C interfere with fertilization and fruit formation and thereby reduce yield^1^. The amount of soil water available to the plants during this time therefore plays an essential role. The projected change in precipitation in July is between −52.5 mm ((≈−67%, REMO) and 36.9 mm (≈47%, HIRHAM5) across all RCMs (Fig. 5, column 3). In the central and north-eastern regions of Germany, the precipitation spatial patterns of different RCMs are similar, while in the north-west and south-east, these patterns differ among RCMs. REMO and RCA3 project a precipitation decrease in almost all regions, although the effect is more pronounced for REMO than for RCA3. RCA3 projects slight increases in precipitation along the German coast, while the pre-Alpine areas face precipitation reductions. RegCM3 and RACMO2 show mixed results.

In all but a few regions, the HIRHAM5-driven mHM simulations show moister conditions in the second climate period, as can be inferred from the soil moisture anomalies in August (Fig. 5, column 4). RegCM3 projects drier soils across the whole country. This trend is also shown by REMO in all areas of Germany, except for the most north-eastern part of the country. RACMO2 and RCA3 show mixed effects in the hydro-meteorological simulations, with more regions expected to become drier. Overall, the model that projects the driest conditions is REMO. For all models, the projected change in the SMI ranges between −0.36 (REMO) and 0.25 (HIRHAM5).

As described above, different spatial patterns and seasonal dynamics are predicted by the RCMs. These patterns can also be seen in the resulting yield changes for the far-future period (Fig. 5, column 5). The blue numbers in the lower right corner of the maps in Fig. 5 show the Spearman rank correlation coefficients of each predictor with the yield anomalies (see here for the mean changes; the coefficients for the respective counties can be found in the Supplementary Fig. S4). We use these correlation coefficients to approximate the effect of the summands from the regression model on the projected yield variability. The summands are the mathematical product of the estimated coefficients for a predictor and the corresponding input data provided by each RCM. As previously described, HIRHAM5 is an exception in regard to changes in yield anomalies and is the only model that projects positive changes (for south-east Germany). For the rest of Germany, low losses of less than 20 dt ha^−1^ a^−1^ in magnitude (≈−4.5%/a) are projected. There, the SMI has the highest correlation coefficient in June. The projections are different for the other RCMs, where losses of up to −57.6 dt ha^−1^ a^−1^ (≈−12.8%/a) are projected. The influence of soil moisture anomalies in June on crop yields seems to be comparatively small. Instead, the temperature in July and soil moisture anomalies in August seem to be the main factors underlying yield anomalies.

Overall, REMO projects the lowest soil moisture anomalies in June and August and the least precipitation in July. However, this model does not represents the greatest loss potential (see Supplementary Table S1). Instead, the greatest loss potential is predicted by RCA3, for which some regions in the east of Central Germany also show high water losses, despite the fact that, compared to other regions, there are no exceptionally extreme temperature, precipitation and soil moisture developments in August (see county-specific correlation coefficients in the Supplementary Fig. S4). The soil moisture factor, in particular, represents a comparatively low soil dryness pressure in this region. However, the losses in this area overlap with regions that become relatively wet in June. This emphasizes that considering soil moisture in multiple months is helpful because wet conditions in June affect yields (Table 1). From this analysis, we conclude that no single driver, such as high temperatures or soil moisture anomalies, defines the total harvest losses; rather, a combination of these sub-seasonal factors must be considered. However, outliers in the projection of yield, as with HIRHAM5, can be traced consistently by evaluating the projected RCM outputs.

## Summary and Conclusion

To our knowledge, this is the first climate impact assessment based on a statistical approach for silage maize yield in Germany as a whole to appear in a peer-reviewed journal. A reduced-form model that considers sub-seasonal soil moisture and meteorological effects was applied. The model is able to explain long-term average changes in yield but is not designed to simulate extreme crop losses in single years. Climate data were derived for two climate periods from five different RCMs for scenario A1B. The maximum absolute projected long-term mean yield loss of silage maize in Germany was estimated to be less than 10% of the average yield between the past and future 30-year periods based on the multi-model RCM simulations driving the mHM and the statistical crop model. Considering adaptation and CO_2_ fertilization, positive yields are expected.

The convergence of process-based and statistical approaches should be further promoted in the near future; the present study took the first step in this process by considering sub-seasonal soil moisture patterns. Further key determinants of plant development need to be integrated into statistical approaches, always based on scientifically sound agronomic knowledge, to address potential multicollinearity problems. An impact assessment of spatial clusters, which better takes the spatial heterogeneity of soils and meteorological dynamics into account, would enable a more precise approach for covering extremes.

Further attention should be paid to improving the precipitation distribution in global climate models. The simulated temperature changes of different global models show the same trends, but precipitation projections, especially the projected seasonal distribution of precipitation, are very different^2,28^. The five RCMs used in the present study have high inter-model variability. For this reason, it is advisable that future research will address such issues through larger RCM ensembles.

## Methods and Data

### Methods

The statistical model developed here is a reduced-form panel approach that exploits the exogenous variation in key explanatory variables^33^. Endogenous variables are not included because they are considered bad control^34^. It incorporates the most influential variables identified in PTMS^4^. The model relates silage maize yield anomalies (Y) to a step-wise function of soil moisture anomalies (SMI) for June and August and polynomials of the demeaned meteorological variables precipitation (P) and temperature (T) for July. The model can be written as:

1 $$\begin{array}{rcl}{Y}_{ik} & = & \sum _{n=1}^{6}\,{\alpha }_{n}{\rm{I}}(SM{I}_{ik}^{June}\in {{\rm{C}}}_{{\rm{n}}})\\  &  & +\,\sum _{j=1}^{3}\,{\beta }_{j}{({{\rm{P}}}_{ik}^{July})}^{j}+\sum _{j=1}^{3}\,{\gamma }_{j}{({{\rm{T}}}_{ik}^{July})}^{j}\\  &  & +\,\sum _{n=1}^{6}\,{\delta }_{n}\,(SM{I}_{ik}^{August}\in {{\rm{C}}}_{{\rm{n}}})\\  &  & +\,c+{\varepsilon }_{ik}\end{array}$$

The observation-specific zero-mean random-error is referred to as *ε*, and *c* is a constant. The *i* index represents the counties within Germany, *k* represents the years, and the superscript *j* represents the degree of the respective polynomial. Polynomials with a degree of three are used according to the results of PTMS^4^. I(⋅) is the indicator function of the soil moisture categories C_n_, where this value is 1 if the SMI belongs to class *n* and 0 otherwise (more details are given below).

As only annual weather deviations from the average of the reference period 1951–2015 are considered by the predictors, the coefficients of the exogenous variables are determined on the basis of inter-annual fluctuations. Farmers are expected to optimize the entire production process at their site based on their experience of local weather conditions. By restricting the coefficients to the same values in all districts, it is implicitly assumed that the response of plants to these inter-annual stressors is the same at all sites. Differences in sensitivity to exogenous weather and soil moisture variations caused by the use of different silage maize varieties or particular soil characteristics are thus ignored by this modelling approach.

### Historical observations

Annual yield data for silage maize are available since 1999 from the Federal Statistical Office of Germany for different district levels^35^. The yield data are not detrended for the period 1999–2015 because no significant linear trend is observed. To obtain anomalies, the mean of each county is subtracted.

The mesoscale Hydrologic Model (mHM) has been used to estimate soil moisture^14,36^. Since silage maize is able to develop a root system that uses the entire root zone depth, a three-layer soil scheme was used to model the soil moisture dynamics over the entire root zone depth (i.e. approximately up to 2 m below ground level)^13^. The soil moisture index (*SMI*) is calculated as a non-parametric and location-specific cumulative distribution function of soil moisture for the period 1951–2015. This procedure enables a comparison across locations^13^. The index ranges between 0 and 1 and quantifies the probability of occurrence of the monthly soil moisture values. For example, a SMI of 0.2 indicates that the soil water saturation fraction is not exceeded during 20% of the time. A median soil moisture value obtains a SMI of 0.5. The advantages of using an index include the relatively low probability of measurement errors and that the estimated coefficients should be less susceptible to attenuation bias^37–39^. In addition, an index minimizes systematic errors associated with spatial data processing and meteorological and climatological modelling^40–44^.

The monthly SMI values are divided into seven classes, following the approach of PTMS^4^. The interval between 0.3 < SMI ≤ 0.7 characterizes normal situations, which are not used in Eq. 1 to avoid perfect multicollinearity in the explaining variables. The lower quantile intervals (SMI ≤ 0.1, 0.1 < SMI ≤ 0.2 and 0.2 < SMI ≤ 0.3) are defined as severe drought, moderate drought and abnormally dry, respectively. Correspondingly, 0.7 < SMI ≤ 0.8, 0.8 < SMI ≤ 0.9 and 0.9 < SMI are defined as abnormally wet, abundantly wet, and severely wet, respectively. All explanatory variables are averaged from their original resolution to the district level to match the spatial scale of the yield data. This averaging weights the explanatory variables according to the area of the non-irrigated agriculture within each grid cell^4^.

Daily precipitation and temperature data are obtained from a station network of the German Weather Service^45^. Interpolation details can be found in Zink *et al*.^46^. All daily values are aggregated to monthly values. By subtracting the county-specific averages, the variables P and T are demeaned. The selected time horizon for P and T is 1951–2015 because this period serves as a basis for generating the SMI. Considering anomalies by either demeaning or employing an index potentially reduces the bias of the coefficients caused by the time-invariant confounding variables specific to each spatial unit for a given period. This approach is not the same as employing fixed effects. However, Lagrange multiplier tests (Honda test for unbalanced panels and F test) show that the remaining fixed effects are insignificant.

### Climate data

The climate data are taken from five RCMs of the EU ENSEMBLES Project for the period 1951–2099^47^. The A1B SRES scenario, which represents a 1.75 °C warming for the period 2046–2065 and a warming of 2.65 °C for the period 2080–2099 compared to the period 1980–1999, is employed^48,49^. The RCMs are forced by the same global model, i.e., the ECHAM5 model of the Max-Planck-Institute for Meteorology in Germany. An earlier meta-analysis showed that impact assessments of crop yields based on ECHAM5 showed lower but positive yield changes than other global models^8^. The applied RCMs are HIRHAM5 by the Danish Meteorological Institute (HIRHAM5), RegCM3 by the Abdus Salam International Center for Theoretical Physics (RegCM3), RACMO2 by the Royal Netherlands Meteorological Institute (RACMO2), REMO by the Max-Planck-Institute for Meteorology (REMO), and RCA3 by the Swedish Meteorological and Hydrological Institute (RCA3). The RCM outputs (i.e., P and T) for the period 1951–2099 are used within this study. The data obtained from these RCMs are also used to drive mHM to simulate soil moisture data. The reference period 1971–2000 is chosen for the climate data. The SMI is thus generated on the basis of the cumulative distribution function of each RCM for this period. Accordingly, the mean value for the period 1971–2000 is subtracted from the meteorological data. Only indices and demeaned input data are used in Eq. 1 to create yield projections. Thus, projections are corrected for bias in the means while preserving the trend. Notably, by using 1971–2000 as the reference period, soil moisture extremes during the periods used for climate projections may lie outside the reference period spectrum. An evaluation showed that this potential effect plays a subordinate role in the analysis. For these extreme values, the SMI is within its bounds (i.e., 0 for dry extremes and 1 for wet ones). The effects of these extreme classes can then be used in the estimation of projected yields.

## Supplementary information

Supplementary Information for “Climate impacts on long-term silage maize yield in Germany”

## Data Availability

All information used in this study has been obtained from the following open sources: Monthly soil moisture data for 1951–2017 are available for Germany on 4 × 4 km^2^ at http://www.ufz.de/export/data/2/209635_SMI_Lall_Gesamtboden_monatlich_1951_2017.zip. Precipitation and temperature data are provided by the German Weather Service - Climate Data Center under ftp://ftp-cdc.dwd.de/pub/CDC/observations_germany/climate/. Regional climate simulations are derived from the ENSEMLES project: http://ensemblesrt3.dmi.dk/. Since 1999 the yield data for the counties are provided by the regional database Germany under https://www.regionalstatistik.de. The data that support the findings of this study are available from the corresponding author upon request.
