# Supplementary material for: Climate impacts on long-term silage maize yield in Germany
Source: Sci Rep. 2019 May 21;9:7674. doi: 10.1038/s41598-019-44126-1 (PMC6529456; doi:10.1038/s41598-019-44126-1)
Supplement: Supplementary file 1 — Supplementary Information for “Climate impacts on long-term silage maize yield in Germany” [file 41598_2019_44126_MOESM1_ESM.pdf]

Supplementary Information for "Climate impacts on long-term silage maize yield in Germany"

Michael Peichl[1*], Stephan Thober[1], Luis Samaniego[1], Bernd Hansjuergens[2], and Andreas Marx[1]**

[1]UFZ-Helmholtz Centre for Environmental Research, Department Computational Hydrosystems, Permoserstrasse 15, D-04318 Leipzig, Germany
[2]UFZ-Helmholtz Centre for Environmental Research, Department Economics, Permoserstrasse 15, D-04318 Leipzig, Germany
[*]michael.peichl@ufz.de
[**]andreas.marx@ufz.de

Figure S1 shows the correlation of soil moisture indices for the months April to October. This indicates the persistence of soil moisture (memory) and the resulting smoother distribution compared to meteorological variables. The SMI in June is strongly correlated with the SMI in the preceding spring. Conversely, August SMI is strongly correlated with the later part of the season, i.e. September and October. This affirms, besides the analysis in Peichl et al., (2018)[1], the choice of SMI for June and August in the statistical model.

The regression model is fitted on a spatiotemporal data set that contains 410 counties and 17 years. All districts with less than nine years of reported yields are excluded from the analysis because the influence of individual observation points is too strong in these cases (see Figure S2). The threshold of 9 was chosen after Cooks' distance[2,3], and the systematic omission of yield data from the 410 counties was evaluated (not shown). There were a total of 286 remaining districts. To allow the evaluation of spatial differences in model performance the county specific quadratic Pearson correlation coefficients between the yield variability of silage maize predicted by the regression model and the observed historical values from the regional statistics for the fitting period 1999–2015 are shown in Fig. S3. It can be stated that the variability is relatively well predicted in the most important maize growing areas in Germany, which are located in the north-western and south-eastern parts of Germany. For regions with high data availability and low correlations, it is worth mentioning that the relative bias between predicted and actual yield is rather small (Fig. 2).

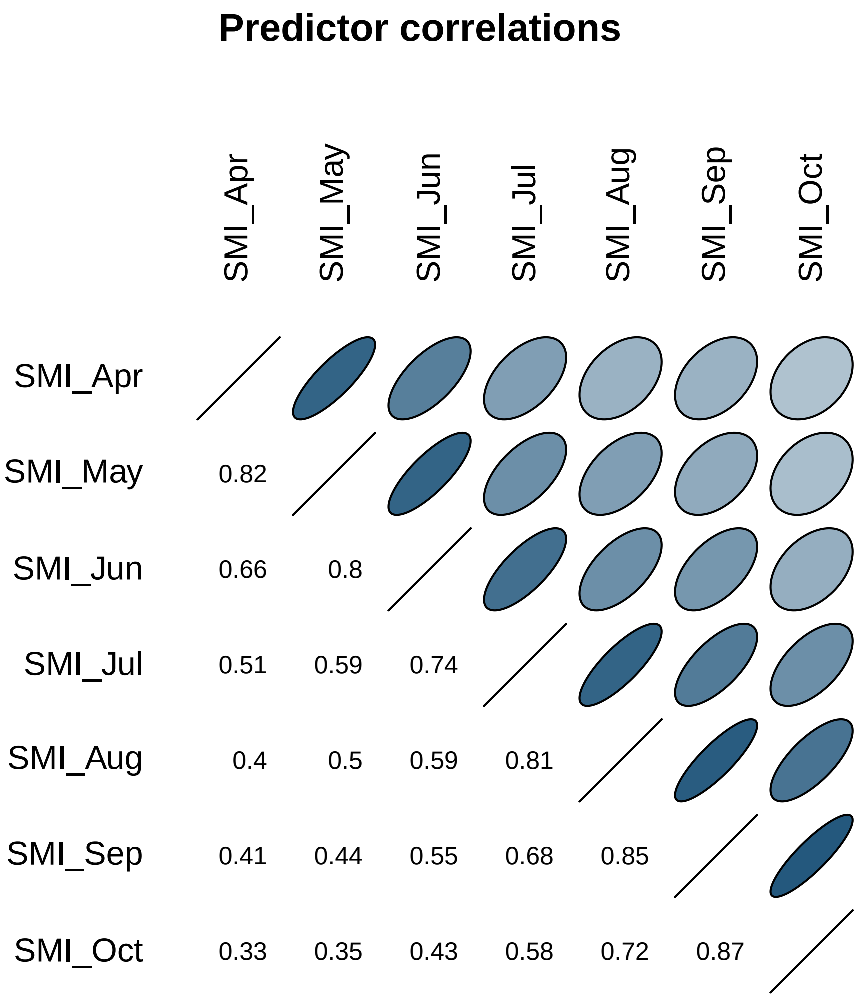

Figure S1. Correlations of soil moisture indexes for the month April to October.

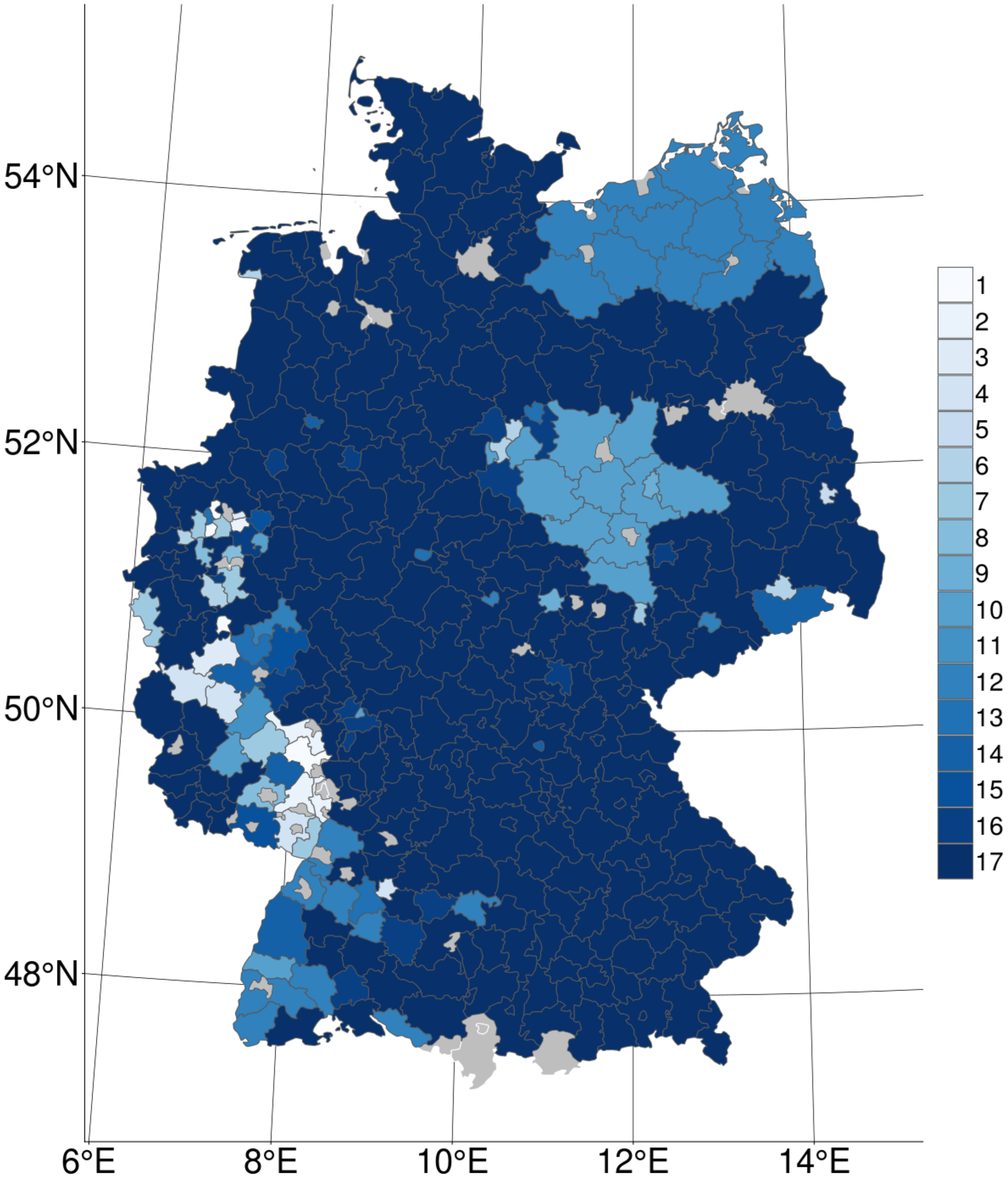

Figure S2. Map showing the number of silage maize yield observations available for each county.

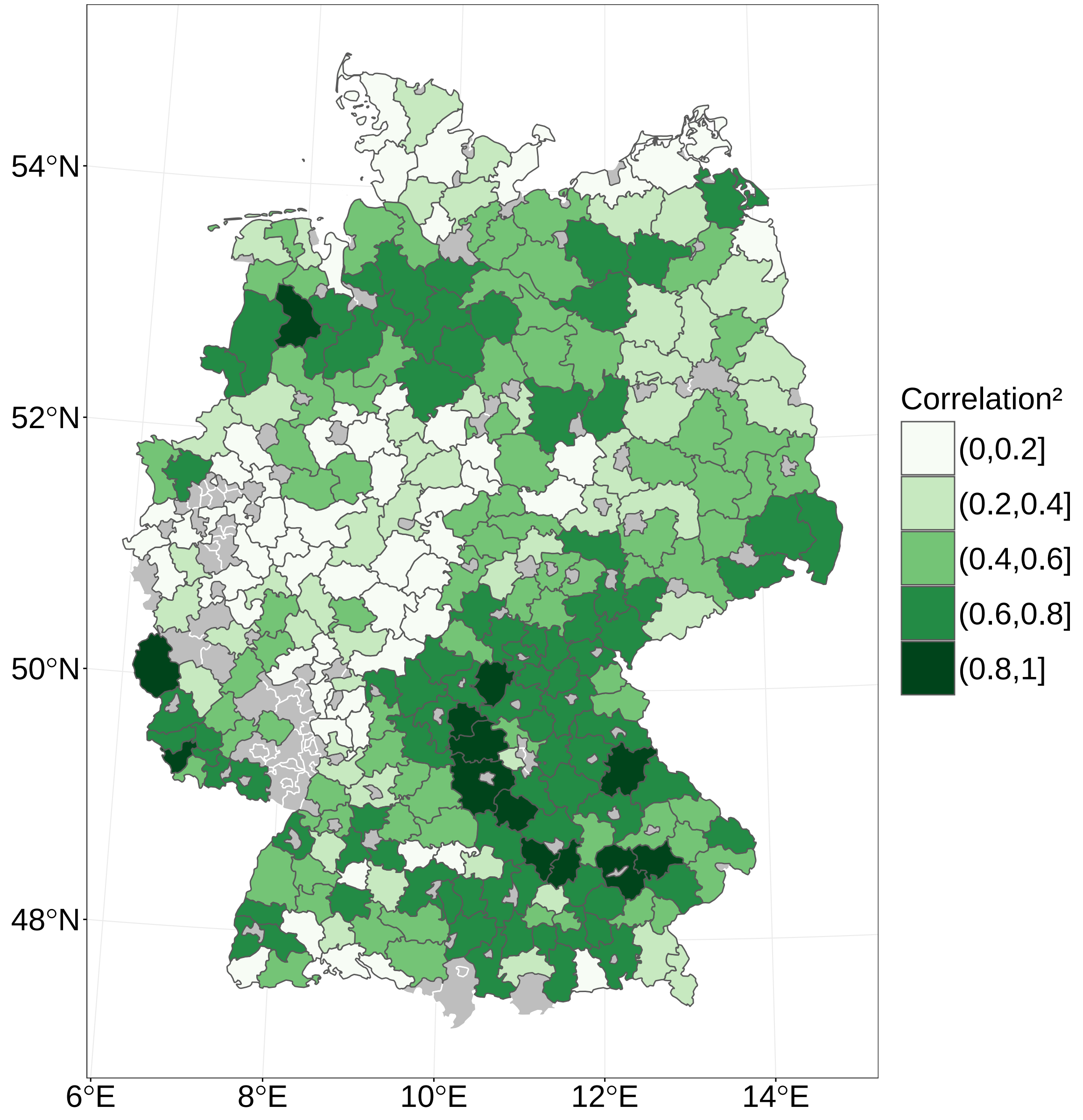

Figure S3. Map showing the county specific quadratic Pearson correlation coefficients between the yield variability of silage maize predicted by the regression model and the observed historical values for the fitting period 1999–2015.

Table S1 shows descriptive statistics of the mean changes in the late climate period 2070–2099 in comparison with the reference period 1971-2000. The data are used in detail in the maps in Fig. 5 (main article). The Spearman correlation coefficients used are those derived from the mean values of each county. The maps in Figure S4 show the Spearman correlation coefficients between the time series of each explanatory variable in the late climate period with the respective time series of the annual yields in each district.

	Statistic	HIRHAM5	RegCM3	RACMO2	REMO	RCA3	historical
June SMI	Mean	0.16	−0.09	−0.02	−0.10	0.02	0.51
	St. Dev.	0.05	0.04	0.06	0.04	0.06	0.27
	Min	0.01	−0.19	−0.18	−0.21	−0.19	0.02
	Max	0.33	−0.01	0.16	0.03	0.18	0.99
Aug. SMI	Mean	0.08	−0.15	−0.11	−0.22	−0.13	0.47
	St. Dev.	0.06	0.05	0.08	0.07	0.08	0.26
	Min	−0.10	−0.29	−0.25	−0.36	−0.33	0.02
	Max	0.25	−0.01	0.12	0.10	0.05	0.99
July P. (mm)	Mean	9.82	0.01	−0.65	−16.56	−8.77	77.80
	St. Dev.	6.79	6.13	7.51	11.10	9.03	39.00
	Min	−9.47	−17.08	−19.54	−52.53	−50.69	1.19
	Max	36.96	19.73	21.54	24.24	11.27	341.00
July T. (°C)	Mean	1.34	2.69	2.98	2.65	2.79	17.70
	St. Dev.	0.26	0.38	0.51	0.50	0.45	1.83
	Min	0.92	1.97	1.91	1.71	1.88	12.60
	Max	1.96	3.51	3.98	3.68	3.76	23.60
Yield ($dt\,dt^{-1}a^{-1}$)	Mean	−4.05	−8.28	−24.26	−34.38	−39.13	447.50
	St. Dev.	6.30	5.35	4.90	7.26	7.52	71.94
	Min	−24.50	−41.75	−36.09	−57.12	−57.62	35.00
	Max	12.37	−10.31	−8.18	−11.17	−17.39	830.00

Table S1. Descriptive statistics of the mean changes in the five RCMs for the climate period 2070-2099 and the climate period 1971-2000. Absolute historical observations are provided for the period 1971-2000 for all RCMs, except for yield (last row). The absolute observed values for the period 1999-2015 are presented in the last row.

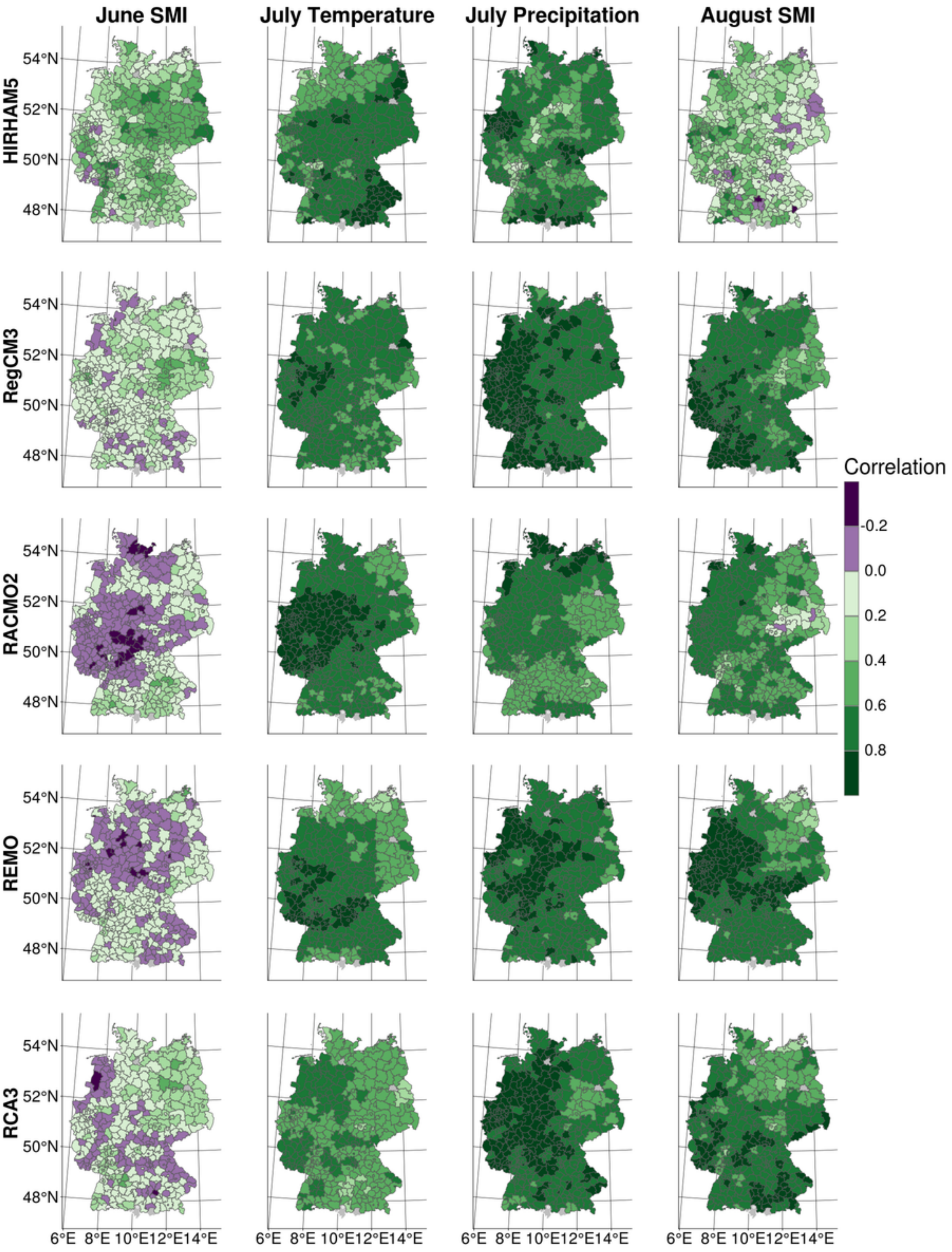

Figure S4. All panels show maps of the Spearman correlation of the summands for each variable (the coefficients used in the model times the input data), with the yield predicted by the model. Data from the climate projections for the period 2070-2099 are used for this purpose. The data are normalized by a procedure that subtracts the mean of the period 1970–2000 from each value. The columns represent the different variables, and the rows represent the RCMs (HIRHAM5, RegCM3, RACMO2, REMO, RCA3).

References

1. Peichl, M., Thober, S., Meyer, V. & Samaniego, L. The Effect of Soil Moisture Anomalies on Maize Yield in Germany. *Nat. Hazards Earth Syst. Sci.* **18**, 889–906, DOI: doi.org/10.5194/nhess-18-889-2018 (2018).
2. Cook, R. D. Detection of Influential Observation in Linear Regression. *Technometrics* **19**, 15–18, DOI: 10.1017/CBO9781107415324.004 (1977).
3. Cook, R. D. Influential Observations in Linear Regression. *J. Am. Stat. Assoc.* **74**, 169–174, DOI: 10.1080/01621459.1979.10481634 (1979).
